# Trauma Patterns and Psychiatric Profiles in Suicide Attempts at a Regional Trauma Center in South Korea: A Retrospective Single-Center Study

**DOI:** 10.3390/jcm14124218

**Published:** 2025-06-13

**Authors:** Young Un Choi, Ji Young Hyun, Seongyup Kim, Keum Seok Bae, Jae Sik Chung, Il Hwan Park, Chan Young Kang, Tae Hui Kim, Chun Sung Byun

**Affiliations:** 1 Department of Surgery, Yonsei University Wonju College of Medicine, Wonju 26426, Republic of Korea; sangorilla@hanmail.net (Y.U.C.); sykimvs@yonsei.ac.kr (S.K.); bksgs@yonsei.ac.kr (K.S.B.); gsjaesik@yonsei.ac.kr (J.S.C.); 2 Trauma Center, Wonju Severance Christian Hospital, Wonju 26426, Republic of Korea; nicecs@yonsei.ac.kr; 3 School of Medicine, CHA University, Seongnam 13496, Republic of Korea; luccy3399@gmail.com (J.Y.H.); chlrhkcy@naver.com (C.Y.K.); 4 Department of Thoracic and Cardiovascular Surgery, Yonsei University Wonju College of Medicine, Wonju 26426, Republic of Korea; 5 Department of Psychiatry, Yonsei University Wonju College of Medicine, Wonju 26426, Republic of Korea; gooddr@yonsei.ac.kr

**Keywords:** suicide attempts, trauma patients, psychiatric diagnoses, emergency department, method of self-harm

## Abstract

**Background**/**Objective**: South Korea continues to have the highest suicide rate among the Organization for Economic Co-operation and Development (OECD) countries, with a growing number of emergency department (ED) admissions related to self-harm and suicide attempts. However, trauma-focused analyses that integrate psychiatric profiles and suicide mechanisms remain limited, hindering effective clinical care and preventive strategies. **Methods**: This retrospective study analyzed trauma patients who presented to the ED of Wonju Severance Christian Hospital following suicide attempts between October 2015 and December 2023. Of 305 self-harm cases, 208 survivors who underwent psychiatric evaluation were included. The variables analyzed included the mechanism, site, and severity of injury (ISS and AIS); psychiatric diagnosis and prior psychiatric history; repeated suicide attempts; alcohol use; physical pain; interpersonal conflict; and economic vulnerability. Chi-square, Fisher’s exact, and Kruskal–Wallis’ tests were used for statistical comparisons. **Results**: Stabbing/cutting (56.7%) and falling (35.6%) were the most common attempts. Mood disorders were the predominant psychiatric diagnosis (63.9%), followed by adjustment disorders (26.0%), alcohol use (25.5%), and psychotic disorders (22.1%). Among the fall-related cases, patients were typically younger and predominantly women. The median ISS was highest in fall-related cases (17) compared with stabbing/cutting (4), with 25.96% of patients with an ISS ≥16, indicating severe trauma. A psychiatric history was associated with a higher incidence of falls (44.3%), and previous suicide attempts correlated with the use of high-lethality attempts. Severe physical pain was linked to stabbing/cutting in 10 of 11 cases. Interpersonal conflict was more frequently associated with stabbing/cutting (59.6%) than with falls (31.9%). No significant association was found between alcohol use and the method of suicide attempt. **Conclusions**: The suicide mechanisms in patients with trauma are closely associated with psychiatric and psychosocial factors. Stabbing/cutting is often impulsive and driven by interpersonal conflict or alcohol use, while falling is more frequent in patients with psychiatric histories of repeated attempts. These findings emphasize the importance of mechanism-informed psychiatric evaluations and trauma protocols. Regionally adapted, interdisciplinary approaches and early psychiatric intervention are crucial for effective post-attempt management and suicide prevention.

## 1. Introduction

Suicide remains a major public health crisis in South Korea. Among the Organization for Economic Co-operation and Development (OECD) member countries, South Korea has the highest suicide rate, and suicide is the leading cause of death among individuals in their teens to their 30s [1]. The number of patients presenting to emergency departments (EDs) due to self-harm or suicide attempts is also increasing [2]. Between 2021 and 2022, nearly 80% of patients injured from self-harm or suicide required emergency medical treatment according to the Korean Triage and Acuity Scale [3]. In 2022, 12,906 individuals died by suicide in South Korea, corresponding to a rate of 25.2 per 100,000 people.

Despite the seriousness of this issue, structured ED-based clinical guidelines and psychiatric post-attempt care programs are limited. This gap is critical given the high recurrence rate of suicidal behavior [4]. For patients presenting to the ED with traumatic injuries from suicide attempts, timely trauma care is essential. Equally important is the identification and treatment of underlying psychiatric conditions to reduce the risk of recurrence.

Identifying the suicide attempt mechanism during the initial ED assessment can help clinicians anticipate likely injury sites, perform repeated assessments, and initiate rapid intervention [5]. Furthermore, recognizing patients at risk for specific suicide methods allows for post-discharge interventions—such as reducing environmental triggers or restricting access to lethal means.

To deliver the appropriate treatment and follow-up care for suicide attempt survivors, it is necessary to understand their psychiatric diagnoses and general characteristics. Analyzing the relationships among suicide mechanisms, injury severity and location, psychiatric diagnoses, psychiatric history, prior suicide attempts, alcohol consumption, severe physical pain, interpersonal conflict before the attempt, and economic vulnerability may allow for the prediction of suicide methods based on patient profiles. Such insights can guide early clinical decisions, reduce missed injuries, and inform the development of more effective relapse prevention strategies.

Accordingly, we aimed to investigate the general characteristics, psychiatric diagnoses, and related clinical and psychosocial factors among patients with trauma who attempted suicide. Our goal was to provide a comprehensive understanding of this patient population to inform ED management and post-attempt care planning.

## 2. Materials and Methods

### 2.1. Inclusion and Exclusion Criteria

We retrospectively reviewed the electronic medical records of patients with trauma who were transported to the ED of Wonju Severance Christian Hospital between 1 October 2015, and 31 December 2023. Patients were included if they presented after a self-harm incident, underwent psychiatric evaluation, and had sufficiently detailed medical records. Patients were excluded if they refused psychiatric consultation, died before psychiatric intervention, used medical methods such as carbon dioxide intoxication, drug administration, or non-cervical hanging, or were dead on arrival.

During the study period, 10,180 patients with trauma were admitted to the ED. Of these, 305 cases involved self-harm or suicide attempts, 158 were related to violence or homicide, and 9717 were unintentional injuries. Among the 305 self-harm/suicide attempt cases, 208 patients survived the initial treatment and underwent psychiatric evaluations. These 208 patients comprised the final study population.

### 2.2. Analysis Variables

The following variables were retrospectively obtained from medical records: patient age, sex, injury intent (intentional vs. unintentional), mechanism of injury, injury location, Abbreviated Injury Scale (AIS) score, Injury Severity Score (ISS), psychiatric diagnosis received after the suicide attempt, alcohol consumption status, prior psychiatric diagnosis, history of physical disability, previous suicide attempts, presence of financial hardship, presence of uncontrolled and severe physical pain contributing to suicidal ideation, and a history of interpersonal conflict or arguments immediately preceding the attempt.

### 2.3. Psychiatric Diagnosis Criteria

To facilitate analysis and interpretation, detailed psychiatric diagnoses were grouped into 18 categories in consultation with board-certified psychiatrists. These categories are listed in Appendix A.

### 2.4. Ethical Approval

This study was approved by the Institutional Review Board (IRB) of Yonsei University Wonju Severance Christian Hospital (IRB No. CR 324050/date: 2 July 2024). The requirement for written informed consent was waived by the IRB due to the retrospective nature of the study and minimal risk to participants. All data were fully anonymized before analysis to ensure confidentiality. This study was not registered in a clinical trial registry, as it is a retrospective observational study using routinely collected clinical data, and registration was not deemed necessary by the IRB.

### 2.5. Statistical Analysis

Variables were compared across groups based on suicide attempt method. Categorical variables are presented as frequencies and percentages, and statistical significance was assessed using the chi-square test. If more than 20% of cells had an expected count < 5, Fisher’s exact test was applied. Continuous variables, which were not normally distributed, are reported as medians and interquartile ranges (IQRs). The Kruskal–Wallis test was used for hypothesis testing of continuous variables, with post hoc analysis conducted using the Bonferroni method. Statistical significance was set at *p* value < 0.05. All statistical analyses were performed using Statistical Analysis System (SAS) software (version 9.4; SAS Institute Incorporated, Cary, NC, USA).

## 3. Results

Among the patients who attempted suicide, the most common method was stabbing (49.52%), followed by falling (35.58%) and cutting (7.21%). Alcohol consumption prior to the attempt was reported in 40% of the cases, and 52% had a documented history of psychiatric illness. Additionally, 32.7% had a prior suicide attempt. Regarding psychiatric diagnoses, mood disorders were the most prevalent (63.9%), followed by adjustment disorders (26.0%), alcohol use disorders (25.5%), and psychotic disorders (22.1%) (Table 1).

When stabbing and cutting were combined into a single category (stabbing/cutting), the distribution of psychiatric diagnoses across all mechanisms remained consistent: mood disorders, adjustment disorders, alcohol use disorders, and psychosis were the most frequently observed. Notably, among patients in the fall group, the proportion of women was relatively higher, and the mean age was lower than in the other groups (Table 2).

In terms of injury severity, the overall mean ISS was 10.4, with 25.96% of patients having an ISS of 16 or higher, indicating that approximately one-quarter sustained severe trauma. Abdominal injuries were the most common (172 cases) and had the highest AIS scores (mean: 1.32). In stabbing/cutting cases, the abdomen was the most frequently injured site, with a median ISS of 4. In contrast, fall-related cases had a median ISS of 17, with injuries most commonly involving the chest, extremities, pelvis, and abdomen (Table 3).

**Figure 1:**
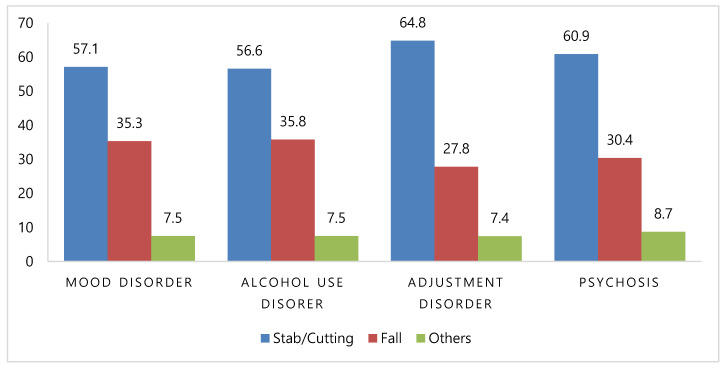
Proportion of suicide mechanisms by main psychiatric diagnosis received after admission.

When the suicide mechanisms were examined across the four most common psychiatric diagnoses, the pattern mirrored that of the overall population: stabbing/cutting was the most frequent, followed by falling and other methods (Figure 1).

We further analyzed the relationship between injury mechanisms and psychiatric diagnoses under various conditions. The overall alcohol consumption rate was 40% (83/208). We investigated whether suicide methods differed by psychiatric diagnosis depending on alcohol consumption; however, no statistically significant differences were found. Among alcohol-consuming patients, the most common psychiatric diagnoses were mood disorders (n = 56), alcohol use disorders (n = 48), and adjustment disorders (n = 26), which did not differ significantly in order or distribution from the non-alcohol-consuming group.

**Figure 2:**
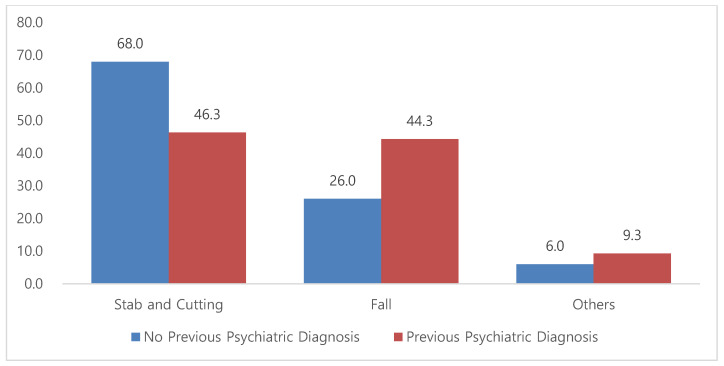
Proportion of suicide mechanisms by previous psychiatric diagnosis (*p* < 0.05).

In patients with a history of psychiatric illness, the distribution of suicide mechanisms shifted: stabbing/cutting accounted for 46.3%, falling for 44.3%, and other mechanisms for 9.3%. Compared with the overall cohort, this group showed a notably higher proportion of falls (Figure 2).

**Figure 3:**
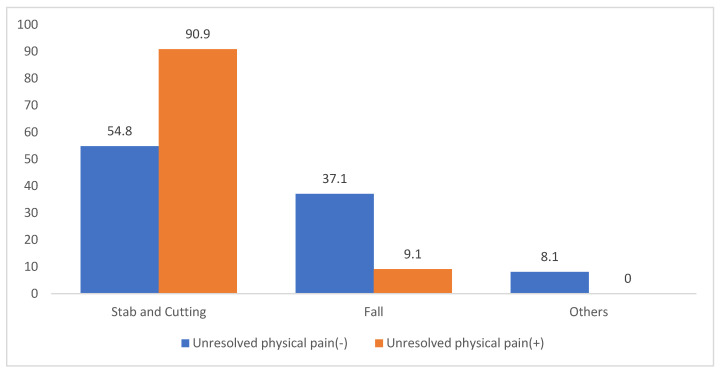
Proportion of suicide mechanisms by severe physical pain (*p* = 0.0948).

Among patients with unresolved severe physical pain, such as that caused by terminal cancer or end-stage lung disease with intractable dyspnea, 10 out of 11 attempted suicide via stabbing or cutting; only 1 patient used falling (Figure 3).

**Figure 4:**
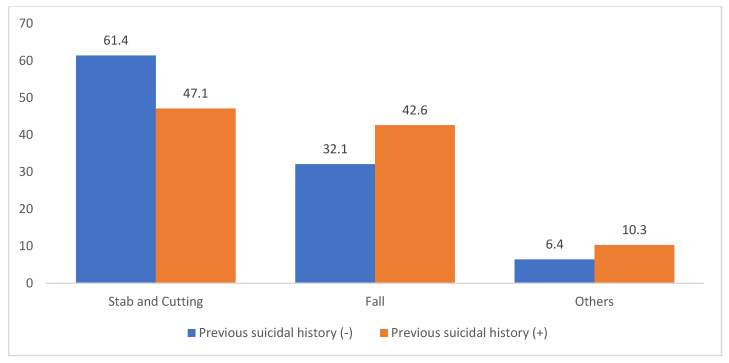
Proportion of suicide mechanisms by previous suicide attempt (*p* = 0.1279).

For those with a previous history of suicide attempts (including drug overdose), the distribution differed from that in those without such a history. In patients without prior attempts, the distribution was as follows: stabbing or cutting (61.4%), falling (32.1%), and other (6.4%). In contrast, among those with prior attempts, the distribution was: stabbing or cutting (47.1%), falling (42.6%), and others (10.3%). This indicates a relative increase in falling, similar to the trend observed in patients with a psychiatric history (Figure 4).

**Figure 5:**
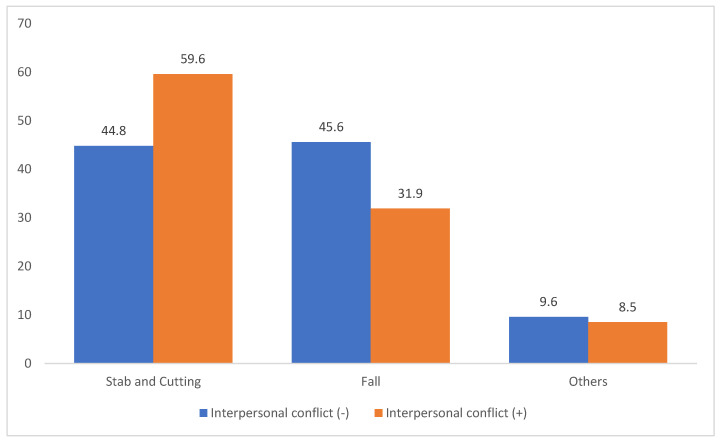
Proportion of suicide mechanisms by conflict with others prior to suicidal attempt (*p* = 0.7869).

Among patients who attempted suicide impulsively following interpersonal conflict, such as fighting with a spouse, friend, or family member, the method distribution also differed. In those without recent conflict, the distribution was: stabbing/cutting (44.8%), falling (45.6%), and others (8.6%). However, in patients who attempted suicide after an argument, the distribution shifted to stabbing or cutting (59.6%), falling (31.9%), and others (8.5%), with stabbing or cutting occurring nearly twice as often as falling (Figure 5).

## 4. Discussion

### 4.1. Trauma Patterns and Psychiatric Profiles in Suicide Attempts

In the present investigation, suicide attempts involving drug intoxication and non-cervical hanging were excluded in order to focus specifically on trauma-related mechanisms. The predominant methods identified were stabbing, cutting, and falling. According to national ED data from 2011 to 2020, excluding drug overdose, the distribution of methods included stabbing (20.4%), suffocation (7.4%), blunt trauma (4.3%), and falling (4.6%). Over the past decade, the proportion of stabbing cases has shown the greatest increase, followed by a steady rise in falls, whereas blunt injuries have decreased. The reported mortality rates for falls and stabbing are 41.6% and 0.6%, respectively [6]. Our findings reflect these national trends. Fall-related injuries were most frequently localized to the thorax, extremities, pelvis, and abdomen. The cohort’s mean ISS was 10.4, with approximately one-quarter of patients presenting with an ISS ≥ 16, predominantly in fall cases. These findings underscore the critical need for clinical vigilance in the early assessment and repeated evaluation of fall-related trauma, particularly given the insidious nature of occult injuries.

Psychiatric diagnosis patterns showed a predominance of mood disorders (70%), followed by adjustment disorders (26%), alcohol use (25%), and psychotic disorders (22%). This distribution was consistent across the mechanisms, with stabbing, falling, and cutting being the most common in descending order. Previous research has shown that individuals with schizophrenia, bipolar disorder, and major depressive disorder are more likely to employ high-lethality methods such as falling (odds ratios [ORs]: 3.38, 3.2, and 2.11, respectively) [7,8]. Our findings align with this literature, though diagnostic grouping into broader categories (e.g., mood vs. psychotic disorders) may have limited the statistical resolution.

### 4.2. Impact of Psychiatric History and Repeated Suicide Attempts

Among the individuals with a documented psychiatric history, the distribution of suicide methods shifted significantly, with falls accounting for 44.4% compared with 26.0% in those without such a history. This shift was especially pronounced among patients with mood and alcohol-related disorders. Although the literature on this topic is limited, Matlach et al. [9] reported that patients with chronic psychiatric illness often engage in impulsive self-harm due to emotional dysregulation or psychotic symptoms, whereas individuals without psychiatric histories tend to exhibit more premeditated behaviors. Furthermore, the individuals with a history of prior attempts in our study also demonstrated a higher propensity for falls (42.6% vs. 32.1%), corroborating the national trends reported by the Korea CDC [2], and findings from international cohort studies suggesting method escalation over time [10].

### 4.3. Influences of Physical Pain, Conflict, and Alcohol on Method Choice

Although previous studies have identified a strong association between chronic physical pain and suicidal ideation [11,12], many have reported overdose as the most common method [13]. However, in this trauma-centered cohort, 10 out of 11 patients with severe chronic pain—primarily those with terminal cancer or end-stage COPD—selected stabbing or cutting as their suicide method. These injuries, while associated with relatively low ISS, were concentrated in high-risk anatomical regions such as the neck, chest, and abdomen. Differences in study design, population characteristics, and the exclusion of drug intoxication cases in our sample may explain the divergence from earlier findings. Given that most of these patients were also diagnosed with mood disorders and supported by the literature highlighting the compounding effects of depression and affective pain in suicidality [14,15], further research with larger sample sizes is warranted to clarify pain-driven suicidal behavior.

Pre-attempt interpersonal conflict was associated with a notable increase in the use of stabbing or cutting (59.6%) compared with falling (31.9%). This finding aligns with research on adolescents in protective environments, where one-third of suicide attempts occur immediately after family disputes and involve readily accessible means [16]. Rimkeviciene et al. [17] reported that impulsive suicide attempts often lack a strong intent to die and are more likely to involve immediately available methods. Consistent with this, our findings showed that the ISS among patients who used stabbing/cutting were all below nine, reinforcing the low-lethality nature of impulsive self-harm.

Alcohol consumption was reported in 40% of the cases; however, no statistically significant associations were found between alcohol use and the choice of suicide method, either within psychiatric diagnostic groups or across the overall cohort. Contrary to studies linking elevated blood alcohol levels to more lethal methods such as hanging or poisoning [18,19], our data indicated a higher incidence of stabbing/cutting among alcohol users. This discrepancy may be due to limitations in self-reported alcohol use and the exclusion of lethal methods such as overdose and non-cervical hanging from our analysis. Previous work by Edwards et al. [20] has shown that alcohol use disorder is associated with the choice of more lethal methods, highlighting the need for objective assessments of alcohol dependence in future studies.

### 4.4. Socioeconomic and Methodological Considerations

Although financial hardship, including poverty and unemployment, did not show a significant relationship with any specific suicide method in this study, prior research during the Greek financial crisis found significant correlations between economic distress and psychiatric diagnoses, such as depressive and anxiety disorders [21]. In our cohort, nearly 70% of economically vulnerable patients were diagnosed with mood disorders, indicating a potential indirect pathway through psychiatric comorbidities rather than a direct effect on method selection.

### 4.5. Integration with Post-Attempt Psychiatric Care and Public Health Strategies

In patients who have attempted suicide, the most important thing to prevent recurrence is physical stabilization [22,23,24]. After this, a psychiatric approach and multifaceted efforts to prevent recurrence are necessary. However, there is currently a lack of periodic psychiatric monitoring in Korea, and this may not effectively prevent the recurrence of suicide attempts. While post-attempt psychiatric care is crucial, current emergency protocols often prioritize physical stabilization over mental health assessments, especially in trauma settings. This study underscores the importance of interventions tailored to suicide mechanisms, psychiatric history, and pain-related factors to prevent recurrence. For example, patients with fall-related injuries could benefit from early psychiatric admission and structured follow-up because of the high lethality and recurrence risk [25].

Standardized suicide risk screening tools, such as the Columbia-Suicide Severity Rating Scale (C-SSRS) or the Patient Health Questionnaire-9 (PHQ-9), should be integrated into trauma care protocols to ensure timely mental health evaluations. Additionally, interdisciplinary collaboration among trauma surgeons, emergency physicians, and psychiatrists should be institutionalized through joint training programs and coordinated care pathways [26,27].

Regional demographic characteristics, such as the high proportion of elderly residents in Gangwon Province, may have influenced both the choice of suicide method and psychiatric diagnosis in this study. Older adults are particularly vulnerable to physical illnesses, social isolation, and economic hardships, all of which are well-documented risk factors for suicide. Public health initiatives involving community outreach, geriatric mental healthcare, and social support tailored to rural and aging populations are essential. Future research using multiregional datasets is needed to better understand these patterns and guide equitable health policies.

### 4.6. Limitations

This study had some limitations. First, psychiatric follow-ups were not systematically conducted at our institution, limiting our ability to assess the long-term mental health outcomes. Second, patients who died at the scene or shortly after arrival, as well as those who used drug intoxication or non-cervical hanging, were excluded, potentially limiting the generalizability of the findings to all suicide attempters. The purpose of this study was to identify and analyze the characteristics of patients with suicidal attempts due to trauma and to determine whether they could be applied in the emergency room. For this reason, deceased patients who could not be analyzed psychiatrically and patients with internal medicine were excluded. Third, the psychiatric diagnoses were categorized into broad groups, despite the likelihood of comorbidities, which may have obscured specific associations with suicide methods. Fourth, the co-occurrence of multiple methods (e.g., wrist-cutting and falling) in individual cases complicates the attribution of a single psychiatric profile. Lastly, the hospital’s location in a region with a disproportionately elderly population may have affected the demographic applicability of these findings.

Despite these limitations, this study provides meaningful insights into trauma-associated suicide attempts and offers a basis for the development of clinical guidelines and targeted prevention strategies. In particular, the mechanism of trauma in patients who attempted suicide and the psychiatric analysis based on it will help us understand the main damaged areas and psychiatric characteristics of patients who attempted suicide and can serve as a cornerstone for preventing recurrence through additional psychiatric analysis.

## 5. Conclusions

This study provides a detailed characterization of patients with trauma who survived attempted suicide, highlighting the interplay between psychiatric diagnoses, injury patterns, and psychosocial factors. Stabbing, cutting, and falling emerged as the most common methods, each associated with distinct clinical and demographic profiles. Patients with a psychiatric history and repeated suicide attempts were more likely to use high-lethality methods such as falling, whereas impulsive attempts triggered by interpersonal conflict or alcohol use were more often associated with lower-lethality mechanisms such as stabbing or cutting.

Mood disorders were the predominant psychiatric diagnosis across all groups, underscoring the need for comprehensive mental health screening and targeted interventions. Additionally, the high prevalence of severe injuries, especially fall-related injuries, emphasizes the necessity for early psychiatric evaluation in trauma care.

While the findings are limited by case selection and setting, this study offers valuable insights into the complex profiles of patients with suicide-related trauma. It supports the integration of psychiatric assessments into trauma protocols and advocates for multidisciplinary collaboration. Future research involving broader, multiregional populations is essential to validate these findings and inform evidence-based, patient-centered strategies for suicide prevention and post-attempt care.

## Figures and Tables

**Table 1:** General characteristics of suicidal patients with trauma.

Variables		Details	Variables		Details
ALL		208	Psychiatric Diagnosis Received After Admission		
Sex	Male	125 (59.1%)			
Age (Years)	Mean ± STD	44.5 ± 18.6	Mood disorder	“Yes”	133 (63.9%)
	Median [IQR]	45.5 [30–57]	Adjustment disorder	“Yes”	54 (26.0%)
	Min, Max	12, 94	Alcohol use disorder	“Yes”	53 (25.5%)
Mechanism	Stab	103 (49.5%)	Psychosis	“Yes”	46 (22.1%)
	Fall	74 (35.6%)	Bipolar disorder	“Yes”	12 (5.8%)
	Cutting	15 (7.2%)	Personality disorder	“Yes”	6 (2.9%)
	Drowning	6 (2.9%)	Organic mental disorder	“Yes”	6 (2.9%)
	Traffic accident	6 (2.9%)	Anxiety disorder	“Yes”	5 (2.4%)
	Hanging	4 (1.9%)	Gambling disorder	“Yes”	4 (1.9%)
Alcohol Use	“Yes”	83 (39.9%)	Obsessive–compulsive disorder	“Yes”	2 (1.0%)
Previous Psychiatric Diagnosis	“Yes”	108 (51.9%)	ADHD	“Yes”	2 (1.0%)
Physical Disorder	“Yes”	72 (34.6%)	PTSD	“Yes”	2 (1.0%)
Previous Suicide Attempt	“Yes”	68 (32.7%)	Intellectual disabilities	“Yes”	2 (1.0%)
Financial Problems	“Yes”	23 (11.6%)	Acute stress disorder	“Yes”	1 (0.5%)
Severe Physical Pain	“Yes”	11 (5.3%)	Conduct disorder	“Yes”	1 (0.5%)
Conflict with Others Prior to Suicide Attempt	“Yes”	47 (22.6%)	Addiction	“Yes”	1 (0.5%)
Number of Psychiatric Diagnosis Received After Admission	1	103 (49.5%)	Tic disorder	“Yes”	1 (0.5%)
	2	86 (41.4%)	Other disorder	“Yes”	1 (0.5%)
	3+	189 (9.1%)			

STD: Standard Deviation. ADHD: Attention Deficit Hyperactivity Disorder. PTSD: Post-Traumatic Stress Disorder.

**Table 2:** Characteristics of participants by suicidal mechanisms.

	Stab or Cutting Injury	Fall	Others	*p*-Value
	N (%)	N (%)	N (%)	
Sex (Female)	39 (33.1)	41 (55.4)	5 (31.3)	0.0065
Age *	48 [37–61]	33.5 [20–48]	48 [40.5–51.5]	<0.0001
Alcohol Use	51 (43.2)	27 (36.5)	5 (31.3)	0.4963
Previous Psychiatric Diagnosis, “Yes”	50 (42.4)	48 (64.9)	10 (62.5)	0.0068
Physical Disorder, “Yes”	50 (42.4)	16 (21.6)	6 (37.5)	0.0128
Previous Suicide Attempt, “Yes”	32 (27.1)	29 (39.2)	7 (43.8)	0.1371
Financial Problem, “Yes”	14 (11.9)	5 (6.8)	4 (25.0)	0.0987
Severe Physical Pain, “Yes”	10 (8.5)	1 (1.4)	0 (0)	0.0948
Conflict with Others Prior to Suicide Attempt, “Yes”	n = 117	n = 72	n = 16	0.8675
	28 (23.9)	15 (20.8)	4 (25.0)	
Psychiatric Diagnosis Received After Admission				
Mood disorder	76 (64.4)	47 (63.5)	10 (62.5)	1
Adjustment disorder	35 (29.7)	15 (20.3)	4 (25.0)	0.3405
Alcohol use disorder	30 (25.4)	19 (25.7)	4 (25.0)	1
Psychosis	28 (23.7)	14 (18.9)	4 (25.0)	0.7305
Bipolar disorder	6 (5.1)	6 (8.1)	0 (0)	0.5691
Personality disorder	1 (0.9)	5 (6.8)	0 (0)	0.0479
Organic mental disorder	5 (4.2)	1 (1.4)	0 (0)	0.6364
Anxiety disorder	3 (2.5)	2 (2.7)	0 (0)	1
Gambling disorder	1 (0.9)	2 (2.7)	1 (6.3)	0.183
Obsessive–compulsive disorder	0 (0)	2 (2.7)	0 (0)	0.2737
ADHD	0 (0)	2 (2.7)	0 (0)	0.2737
PTSD	0 (0)	2 (2.7)	0 (0)	0.2737
Intellectual disabilities	2 (1.7)	0 (0)	0 (0)	0.5944
Acute stress disorder	0 (0)	1 (1.4)	0 (0)	0.4327
Conduct disorder	0 (0)	1 (1.4)	0 (0)	0.4327
Addiction	1 (0.9)	0 (0)	0 (0)	1
Tic disorder	0 (0)	1 (1.4)	0 (0)	0.2737
Other disorder	1 (0.9)	0 (0)	0 (0)	1

* Median [IQR]. IQR: Interquartile Range. ADHD: Attention Deficit Hyperactivity Disorder. PTSD: Post-Traumatic Stress Disorder.

**Table 3:** Injury score by suicidal mechanisms.

	Stab/ Cutting		Fall		Others		ALL		
	N	%	N	%	N	%	N	%	*p*-Value
Head and Neck									
Injury (AIS score 1+)	31	26.27	30	40.54	8	50	69	33.17	0.041
Severe Injury (AIS score 3+)	6	5.08	9	12.16	3	18.75	18	8.65	0.049
Face									
Injury (AIS score 1+)	6	5.08	24	32.43	6	37.5	36	17.31	<0.001
Severe Injury (AIS score 3+)	0	0	2	2.7	0	0	2	0.96	0.126
Thorax									
Injury (AIS score 1+)	13	11.02	50	67.57	6	37.5	69	33.17	<0.001
Severe Injury (AIS score 3+)	8	6.78	32	43.24	3	18.75	43	20.67	<0.001
Abdomen									
Injury (AIS score 1+)	81	68.64	45	60.81	4	25	130	62.5	0.003
Severe Injury (AIS score 3+)	22	18.64	20	27.03	0	0	42	20.19	0.030
Extremities and Pelvis									
Injury (AIS score 1+)	30	25.42	61	82.43	8	50	99	47.6	<0.001
Severe Injury (AIS score 3+)	10	8.47	19	25.68	0	0	29	13.94	<0.001
MAIS									
Median [IQR]	2	[1–3]	3	[2–4]	2	[1–3]	2	[1–3]	<0.001
Severe Injury (AIS score 3+)	41	34.75	52	70.27	6	37.5	99	47.6	<0.001
ISS									
Median [IQR]	4	[1–9]	17	[10–24]	6.5	[2–10.5]	9	[3–16]	<0.001
Severe Injury (ISS 16+)	11	9.32	40	54.05	3	18.75	54	25.96	<0.001

AIS: Abbreviated Injury Scale. ISS: Injury Severity Score. MAIS: Maximum AIS. IQR: Interquartile Range.

## Data Availability

The datasets used and/or analyzed in the current study are available from the corresponding author upon reasonable request.

## Abbreviations

The following abbreviations are used in this manuscript:

COPD	Chronic Obstructive Pulmonary Disease
ISS	Injury Severity Score
C-SSRS	Columbia-Suicide Severity Rating Scale
PHQ-9	Patient Health Questionnaire-9

## Appendix A

**Table A1:** Psychiatric criteria and diagnosis.

Criteria	Psychiatric Diagnosis
1. Personality disorder	Antisocial personality disorder Borderline personality disorder Cluster B personality disorder Paranoid personality disorder Schizotypal personality disorder
2. Organic mental disorder	Delirium due to another medical condition, acute, hyperactive Delirium, hyperactive Frontal lobe syndrome Impulse control disorder Unspecified dementia Vascular dementia
3. Mood disorder	Depression Depression, anxiety disorder, psychosis NOS Depression, NOS Depressive disorder, recurrent episode, severe Depressive disorder, unspecified Dysthymia MDD Other specified depressive disorder PDD Persistent depressive disorder Persistent depressive disorder, early onset, severe
4. Alcohol use disorder	Alcohol use disorder, Problematic pattern of alcohol use
5. Obsessive–Compulsive Disorder	-
6. Adjustment disorder	-
7. Psychosis	Brief psychotic disorder, w/marked stressor w/perplexion Major depressive disorder with mood-congruent psychotic feature Other specified schizophrenia spectrum and other psychotic disorder Other specified SPR or psychotic disorder R/O depressive disorder, with psychotic feature R/O MDD, recurrent episode, severe, with psychotic feature R/O MDD, with PFS R/O steroid-induced psychosis Schizoaffective disorder Schizoaffective disorder, bipolar type Schizophrenia Schizophreniform disorder Unspecified catatonia
8. Bipolar disorder	Bipolar affective disorder Bipolar I disorder Bipolar disorder, MDD Bipolar I disorder, with mood-congruent psychotic features Other specified bipolar and related disorder
9. ADHD	-
10. Anxiety disorder	Anxiety disorder Panic disorder
11. Gambling disorder	-
12. Acute stress disorder	-
13. Intellectual disabilities	-
14. Conduct disorder	-
15. PTSD	-
16. Addiction	Substance use disorder, hypnotics or sedatives
17. TIC disorder	-
18. Other disorder	Personal history of self–harm
